# Comparative study of relapses in Severe Mental Disorder among the different long-acting injectable treatments available on the market

**DOI:** 10.1192/j.eurpsy.2025.2062

**Published:** 2025-08-26

**Authors:** P. Andres-Olivera, F. Prieto-Flores, J. de la Iglesia, E. Dominguez-Alvarez, C. P. Rodriguez, C. Munaiz-Cossio, R. K. Gonzalez-Bolaños, R. Brito-Rey, C. Marín-Lorenzo

**Affiliations:** 1 Psichiatry, CAUSA; 2 Medicine; 3 USAL; 4 CAUSA, Salamanca, Spain

## Abstract

**Introduction:**

Schizophrenia affects approximately 1% of the population aIts treatment is mainly based on antipsychotics, although therapeutic non-compliance is common due to lack of illness insight and side effects. Long-acting injectable antipsychotics emerged as an alternative to improve treatment adherence. This study investigates relapses in patients with severe mental illnesses treated with long-acting injectable antipsychotics, originating from the province of Salamanca.

**Objectives:**

To evaluate and compare the efficacy of different long-acting injectable treatments available in the market in preventing relapses in patients with Severe Mental Disorder (SMD), through retrospective analysis of epidemiological, clinical, and treatment data obtained from electronic medical records.

**Methods:**

This is an observational, retrospective, and comparative study using anonymized data extracted from electronic medical records of patients diagnosed with Severe Mental Disorder (SMD) who have been treated with Long-Acting Injectable (LAI) medications. The study period covers from January 2018 to December 2022.

**Results:**

The study contains information from 161 patients, with a uniform distribution by age and sex. The main group presents psychotic disorders (74.5%), followed by bipolar disorder (18%). Monthly Long Acting aripiprazole is the most used injectable antipsychotic (39.8%). Side effects were recorded, such as extrapyramidal symptoms (11.9%) and sexual dysfunction (8.8%). Antipsychotic switching occurred in 19.5% of patients. The absence of relapses was higher for six-month long-acting paliperidone palmitate (80%) and lower for Monthly Long Acting aripiprazole (69.4%), a survival analysis was performed using the Kaplan-Meier method.

**Conclusions:**

The comparative study reflects that, although Abilify Maintena was the most used, no significant differences were found in relapse prevention among different treatments. Survival analysis also did not yield conclusive results. Although the study has some limitations, such as a small sample size and missing data in some medical records, it provides a starting point for future research.Despite the limitations of this study due to the small sample size and lack of statistically significant results regarding the efficacy of injectable antipsychotics, the study provides information on the use of these treatments in patients with Severe Mental Disorder. Although Monthly Long Acting aripiprazole was the most used, side effects do not seem to be related to its efficacy. The results suggest that there are no significant differences between long-acting injectable antipsychotics available in the market. However, it is important to note the significance of this research topic for the future, given its clinical implications.

**Disclosure of Interest:**

None Declared

